# Tissue-Adhesive and Biocompatible Zein-Polyaniline-Based Hydrogels for Mechanoresponsive Energy-Harvesting Applications

**DOI:** 10.3390/gels11050307

**Published:** 2025-04-22

**Authors:** Maduru Suneetha, Seainn Bang, Sarah A. Alshehri, Sung Soo Han

**Affiliations:** 1 School of Chemical Engineering, Yeungnam University, 280 Daehak-Ro, Gyeongsan 38541, Gyeongbuk, Republic of Korea; seainn13@naver.com; 2 Department of Chemistry, College of Science, Princess Nourah bint Abdulrahman University, P.O. Box 84428, Riyadh 11671, Saudi Arabia; saalshehry@pnu.edu.sa; 3 Research Institute of Cell Culture, Yeungnam University, 280 Daehak-Ro, Gyeongsan 38541, Gyeongbuk, Republic of Korea

**Keywords:** tissue-adhesive hydrogels, zein-polyaniline, mechanoresponsive energy harvesting, wearable strain sensors

## Abstract

Flexible, biocompatible, and adhesive materials are vital for wearable strain sensors in bioelectronics. This study presents zein-polyaniline (ZPANI) hydrogels with mechanoresponsive energy-harvesting properties. SEM revealed a sheet-like fibrous morphology, enhancing adhesion. Incorporating 0.5 wt% polyaniline (PANI) introduced nanostructured aggregates, while higher PANI concentrations (3–5 wt%) formed intertwined fibrous networks, improving the mechanical integrity, surface area, and conductivity. PANI enhanced electrical conductivity, and the hydrogels displayed excellent swelling behavior, ensuring flexibility and strong tissue adhesion. Biocompatibility was validated through fibroblast cell culture assays, and the adhesive properties were tested on substrates, such as porcine skin, steel, and aluminum, demonstrating versatile adhesion. The adhesion strength of hydrogels to porcine skin was greatly enhanced with an increasing amount of PANI. The maximum adhesion strength was found to be 30.1 ± 2.1 kPa for ZPANI-5.0. Mechanical testing showed a trade-off between strength and conductivity. The tensile strength decreased from 13.4 kPa (ZPANI-0) to 7.1 kPa (ZPANI-5.0), and the compressive strength declined from 18.5 kPa to 1.6 kPa, indicating increased brittleness. A rheological analysis revealed enhanced strain tolerance (>500% strain) with an increasing PANI content. The storage modulus (G′) remained stable up to 100% strain in PANI-free hydrogels but collapsed beyond 450% strain, while PANI-containing hydrogels exhibited improved viscoelasticity. Mechanical testing showed robust voltage output signals under compression within a 20 s response time. Despite the reduced mechanical strength, energy-harvesting tests showed a surface power density of 0.12 nW cm^−2^, charge storage of 0.71 nJ, and a surface energy density of 1.4 pWh cm^−2^. The synergy of the piezoelectric response, bioadhesion, and tunable viscoelasticity establishes ZPANI hydrogels as promising candidates for wearable sensors and energy-harvesting applications. Optimizing the PANI content is crucial for balancing mechanical stability, adhesion, and electrical performance, ensuring long-term bioelectronic functionality.

## 1. Introduction

The demand for flexible, biocompatible, and responsive materials in wearable electronics has significantly increased over the past few decades, particularly for applications in healthcare monitoring [1,2,3]. With advancements in bioelectronics, there is a growing need for materials that not only offer high sensitivity but also exhibit flexibility, mechanical durability, and energy-harvesting capabilities [3]. Such materials can be integrated into wearable devices to monitor various physiological parameters, providing real-time data for medical diagnostics and health management. As wearable bioelectronics continue to evolve, particularly in the fields of health monitoring, human–computer interaction, and rehabilitation, the development of strain sensors that can accurately measure deformations in soft tissues, muscles, and skin is of utmost importance [4,5]. These strain sensors need to possess several key characteristics, including high sensitivity to small mechanical changes, robust durability, and the ability to function efficiently under dynamic, often harsh, real-life conditions.

Hydrogels, a class of water-swollen, cross-linked polymer networks, have garnered considerable attention in recent years for their potential in a variety of applications, especially in wearable sensors [6,7,8,9]. Hydrogels are particularly attractive because of their unique combination of high water content, flexibility, and biocompatibility, which makes them ideal candidates for integration into flexible wearable devices [10]. Additionally, hydrogels exhibit tunable mechanical properties, such as elasticity and viscoelasticity, which can be optimized for specific applications. The soft, rubbery nature of hydrogels is particularly advantageous when designing strain sensors that need to conform to skin surfaces or other soft tissues [11]. The flexibility of these materials allows them to accommodate the natural movement of the body, making them ideal for wearable healthcare applications where monitoring movement or strain is critical.

Natural polymers, such as zein, have emerged as a promising choice for hydrogel development due to their inherent biocompatibility, biodegradability, and ability to form stable networks under mild conditions [12,13,14]. Zein, a plant-derived protein extracted from corn, has been widely used in the preparation of hydrogels for various biomedical applications, including drug delivery and tissue engineering. Its biocompatibility and non-toxic nature make it particularly suitable for integration with the human body [13,15,16]. However, one limitation of zein-based hydrogels is their relatively low electrical conductivity, which can hinder their performance in electronic applications, particularly those that require the sensing of mechanical deformations, such as strain sensors. To overcome this limitation, the incorporation of conductive materials, such as polyaniline (PANI), into the hydrogel matrix is an attractive strategy.

Polyaniline (PANI), a conductive polymer, is widely recognized for its ease of synthesis, tunable conductivity, and environmental stability, making it an ideal candidate for enhancing the electrical properties of hydrogels [17]. Its unique conjugated backbone structure allows for efficient charge transport, which is further modulated by its doping and de-doping behavior. Through protonation or oxidation states, PANI can transition among insulating (leucoemeraldine), semiconducting (emeraldine base), and highly conductive (emeraldine salt) states, enabling dynamic electrical responsiveness. This property is particularly advantageous for bioelectronic applications where conductivity needs to be finely controlled based on external stimuli, such as pH changes, applied electric fields, or mechanical deformation. The integration of PANI with biopolymers like zein is especially beneficial as it imparts electrical conductivity without compromising the biocompatibility, flexibility, or structural integrity [18]. Moreover, the synergistic interaction between PANI and the biopolymer matrix not only enhances conductivity but also strengthens the hydrogel’s mechanical properties. The incorporation of PANI into zein-based hydrogels significantly improves stretchability, durability, and resistance to mechanical fatigue, making them highly suitable for applications in strain sensing and real-time physiological monitoring. The ability of PANI-based hydrogels to exhibit piezoresistive behavior—where conductivity varies with applied strain—renders them ideal for wearable bioelectronic devices, enabling the precise detection of muscle contractions, joint movements, and other biomechanical activities. By leveraging the conductive and mechanically adaptive nature of PANI, these hydrogels provide a robust and versatile platform for next-generation biomedical and bioelectronic applications.

In this study, we introduce a novel class of tissue-adhesive hydrogels made from zein and polyaniline (ZPANI). These hydrogels are designed to combine the high electrical conductivity of PANI with the biocompatibility, flexibility, and strong tissue-adhesion properties of zein. The incorporation of PANI within the hydrogel matrix not only enhances its electrical conductivity but also reinforces its mechanical strength, allowing it to endure significant strains while maintaining flexibility. The formation of ZPANI hydrogels involves multiple non-covalent and covalent interactions that contribute to their structural integrity and functional performance. Hydrogen bonding between the hydroxyl and amide groups of zein and the imine groups of PANI plays a crucial role in stabilizing the network. Electrostatic interactions between the positively charged polyaniline chains and the negatively charged functional groups within zein further enhance cohesion within the hydrogel matrix. Additionally, hydrophobic interactions among the nonpolar domains of zein facilitate self-assembly, contributing to the hydrogel’s mechanical robustness. The presence of π–π stacking interactions between the aromatic rings of PANI and zein further reinforces the structural framework. These synergistic interactions not only contribute to the gel’s stability but also enhance its tissue-adhesion properties, allowing it to firmly adhere to various substrates, including biological tissues. This strong adhesion is particularly beneficial for applications in the real-time monitoring of physiological movements, such as muscle contractions, joint flexion, and other body motions, making ZPANI hydrogels a promising candidate for next-generation wearable bioelectronic devices.

## 2. Results and Discussion

### 2.1. Formation of Hydrogels

Firstly, PANI was prepared using APS-induced polymerization. An SEM analysis revealed a fibrillar and porous morphology with interconnected networks, favorable for charge transport and conductivity enhancement (Figure S1). The uniform polymerization, aided by APS and HCl, ensured structural integrity and consistent fibril formation. This morphology makes PANI highly suitable for applications in sensors, supercapacitors, and conductive hydrogels, where a high surface area and efficient charge transport are critical.

**Figure 1:**
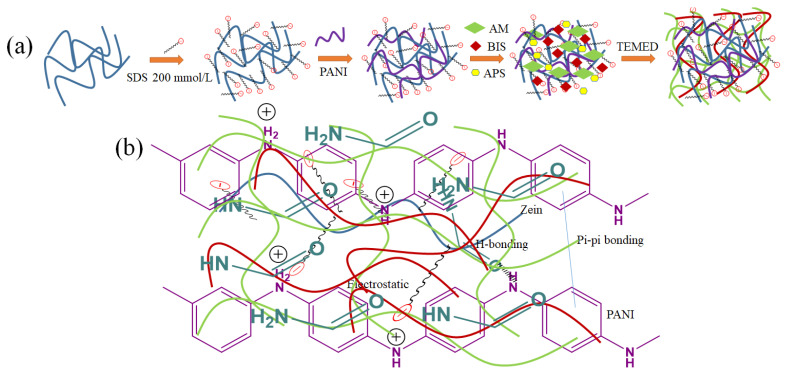
(**a**) Illustration of the hydrogel synthesis process, and (**b**) highlighting the molecular interactions and crosslinking mechanisms of ZPANI hydrogel.

The formation of ZPANI composite hydrogels involves a multi-step process that includes the polymerization and crosslinking of acrylamide, combined with the incorporation of zein and PANI into a stable three-dimensional network (Figure 1). Initially, zein is dissolved in SDS, which aids its solubility and dispersion. PANI is then added at varying concentrations to modify the hydrogel’s electrical and mechanical properties. AM is introduced as the monomer for polymerization, and MBA serves as a crosslinking agent to create covalent bonds between polymer chains. APS initiates the free radical polymerization of AM, and TEMED accelerates the process. The mixture is poured into molds and allowed to gel at room temperature, followed by a stabilization period. At lower concentrations of PANI (up to 3 wt%), the polymer interacts with the zein matrix and acrylamide chains to enhance the hydrogel’s properties, but at higher concentrations (above 3 wt%), excessive viscosity disrupts mixing and polymerization, leading to incomplete gelation. The hydrogels are categorized based on the PANI content (ZPANI-0, ZPANI-0.5, ZPANI-1.0, ZPANI-3.0, and ZPANI-5.0). While higher PANI concentrations improve electrical conductivity, they compromise the gelation efficiency, resulting in poor hydrogel formation. In conclusion, the ZPANI composite hydrogels demonstrate enhanced mechanical, conductive, and biocompatible properties, making them suitable for applications in tissue engineering and wearable sensors, although excessive PANI disrupts the gelation process. However, at higher concentrations of PANI (greater than 5 wt%), the hydrogel formation is disrupted due to the high viscosity of the solution, which impedes the proper mixing and homogeneity required for effective crosslinking. Excessive PANI concentrations lead to interference with both ionic and covalent bonding during the polymerization, resulting in poor gelation and an incomplete crosslinked structure. As a result, the formation of a well-structured hydrogel network is hindered, preventing the formation of stable hydrogels at higher PANI concentrations. Therefore, moderate levels of PANI (up to 5 wt%) enhance the mechanical and conductive properties of the hydrogel, while higher concentrations disrupt the gelation process, compromising the overall structure and performance of the hydrogel. APS can interfere with PANI during hydrogel formation, impacting the polyacrylamide network and mechanical properties. APS acts as both a redox initiator for acrylamide polymerization and an oxidizing agent for PANI, which reduces the availability of free radicals for crosslinking, leading to a weaker and less dense hydrogel matrix. This interference decreases the compressive and tensile strength due to reduced crosslinking density and the rigid, disruptive nature of PANI particles, while increasing the tensile strain due to greater network flexibility. To mitigate these effects, strategies, such as optimizing the APS concentration, sequential polymerization, using alternative initiators, or stabilizing PANI dispersion, can help to improve hydrogel uniformity and mechanical performance.

### 2.2. Rheological Properties of Strain Amplitude for ZPANI Hydrogels

**Figure 2:**
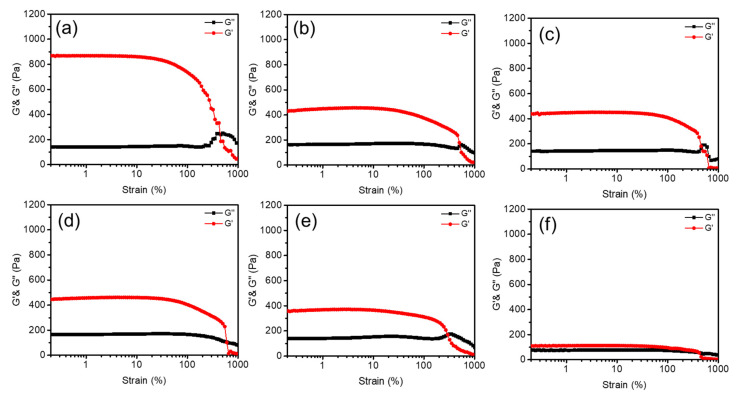
Rheological analysis of ZPANI hydrogels with varying polyaniline (PANI) contents (**a**) 0, (**b**) 0.5, (**c**) 1.0, (**d**) 3.0, (**e**) 5.0 and (**f**) 7.0 wt% under strain amplitude, illustrating the storage modulus (G’) and loss modulus (G”).

The rheological properties of ZPANI hydrogels were studied under strain amplitude, focusing on the effects of varying PANI contents (0, 0.5, 1, 3, 5, and 7 wt%) (Figure 2). The storage modulus (G’), loss modulus (G”), and loss factor (tan δ) provided insights into the mechanical stability and viscoelastic behavior of the hydrogels. As observed in the figure, for the hydrogels without PANI (0 wt%), the G’ and G” remained constant up to a 100% strain, indicating structural stability within this range. However, at a 450% strain, the hydrogel network collapsed, as evidenced by a sharp decline in the G’, suggesting the limits of its elastic behavior. In contrast, hydrogels with PANI (0.5–7 wt%) demonstrated greater structural stability, with G’ and G” crossover points occurring beyond a 500% strain. This enhanced strain tolerance can be attributed to the reinforcement provided by PANI, which resists network deformation and maintains mechanical integrity under higher strain.

**Figure 3:**
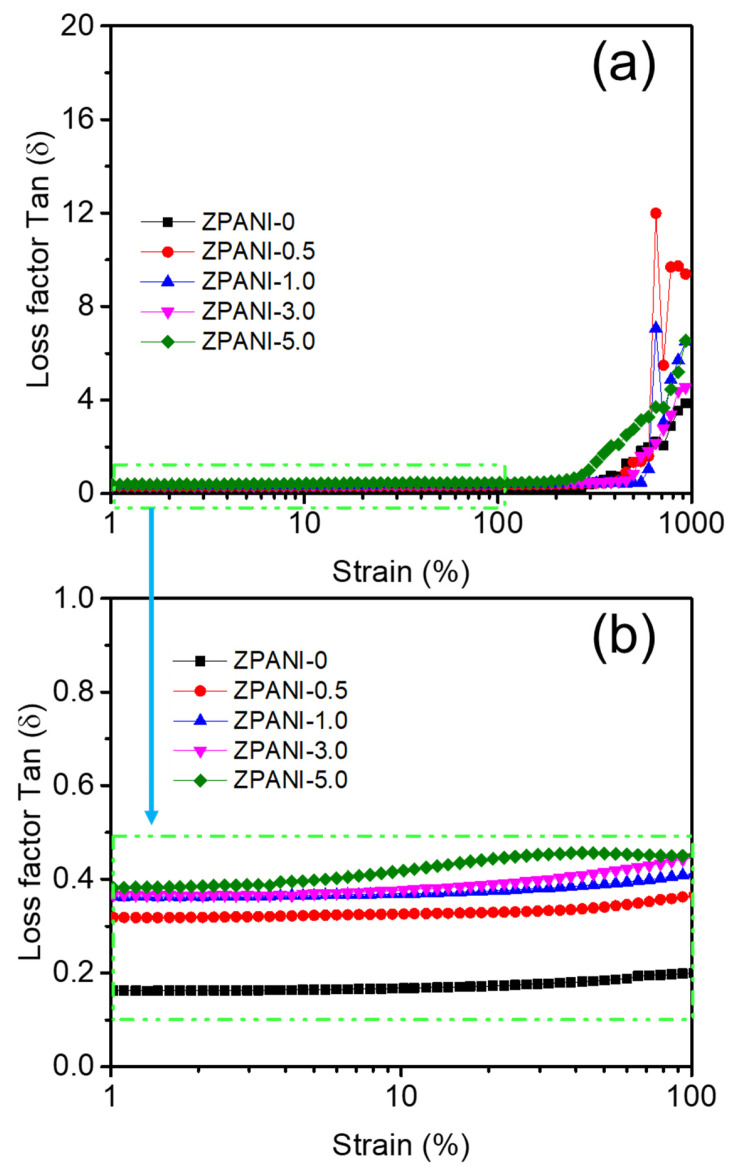
Variation in the loss factor (Tan δ) with the polyaniline (PANI) content (0, 0.5, 1.0, 3.0, and 5.0 wt%) in ZPANI hydrogels (**a**) 1–1000 strain%, and (**b**) 1–100 strain (%).

The loss factor (tan δ), representing the ratio of viscous-to-elastic behavior, increased with the PANI content (Figure 3). With 0 wt% PANI, tan δ was 0.15, indicative of predominantly elastic behavior. With increasing PANI concentrations, tan δ rose to 0.32 (0.5 wt%), 0.37 (1 wt%), 0.38 (3 wt%), and 0.42 (5 wt%), reflecting a gradual shift towards more viscous characteristics. At 7 wt% PANI, tan δ reached 0.86, indicating a significantly weakened structure dominated by viscous behavior. The decreasing G’ and increasing tan δ with higher PANI contents suggest that PANI disrupts the crosslinked PAAm network. Ammonium persulfate, used as an initiator, may preferentially participate in PANI polymerization rather than acrylamide crosslinking, reducing the network’s stiffness. Although hydrogels with higher PANI contents exhibited enhanced strain tolerance (>500% strain), their overall elastic modulus diminished, and viscosity increased. The results indicate a trade-off between mechanical stability and viscoelastic properties with varying PANI contents. Lower PANI concentrations (0.5–5 wt%) maintain a balanced network structure with moderate elasticity and strain tolerance. However, at higher concentrations (7 wt%), the hydrogels become softer and more viscous, with limited structural integrity. This behavior suggests potential applications in areas requiring high deformability and energy dissipation but highlights the need for optimizing the PANI content to achieve the desired mechanical properties. Thus, the incorporation of PANI significantly influences the rheological performance of ZPANI hydrogels, with an increasing PANI content enhancing strain tolerance at the cost of elasticity and structural robustness.

### 2.3. Mechanical Properties

**Figure 4:**
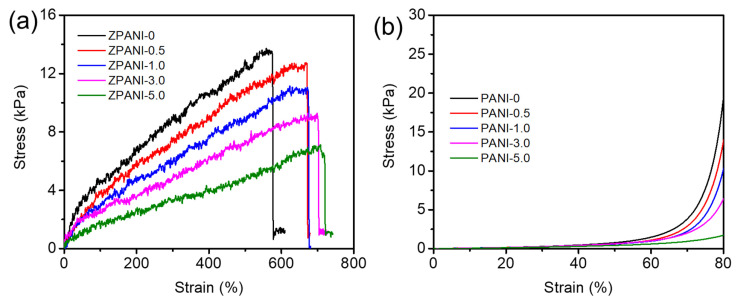
(**a**) Tensile stress–strain curves for ZPANI hydrogels and (**b**) compressive properties of ZPANI hydrogels.

The mechanical behavior of zein–polyacrylamide hydrogels reinforced with varying amounts of polyaniline (PANI) was evaluated in terms of tensile and compressive strengths (Figure 4). The maximum tensile strength of the hydrogels decreased with an increasing PANI content. ZPANI-0 (0% PANI) exhibited the highest tensile strength at 13.4 kPa, while the tensile strengths of ZPANI-0.5 (0.5% PANI), ZPANI-1.0 (1.0% PANI), ZPANI-3.0 (3.0% PANI), and ZPANI-5.0 (5.0% PANI) decreased to 12.7 kPa, 10.9 kPa, 9.2 kPa, and 7.1 kPa, respectively. This suggests that while PANI enhances electrical conductivity, it may reduce the mechanical integrity, likely due to incompatibility between PANI and the zein–polyacrylamide matrix at higher concentrations. The compressive strength of the hydrogels at an 80% strain also decreased with an increasing PANI content. ZPANI-0 exhibited the highest compressive strength at 18.5 kPa, while compressive strengths of ZPANI-0.5, ZPANI-1.0, ZPANI-3.0, and ZPANI-5.0 decreased to 13.8 kPa, 10.6 kPa, 6.4 kPa, and 1.6 kPa, respectively. This significant reduction in compressive strength indicates that higher PANI concentrations may cause the hydrogel network to become more brittle, reducing its ability to withstand compressive forces.

The decrease in both tensile and compressive strengths with increasing PANI contents highlights a trade-off between enhancing electrical conductivity and maintaining mechanical strength. While PANI improves the electrical properties of the hydrogel, it compromises its mechanical performance at higher concentrations, a common phenomenon in polymer composites with conductive fillers. Despite the reduction in mechanical strength, the improved electrical conductivity and the potential for mechanoresponsive applications, such as strain sensing and energy harvesting, make these hydrogels promising candidates for bioelectronics.

### 2.4. Structural Characterization

**Figure 5:**
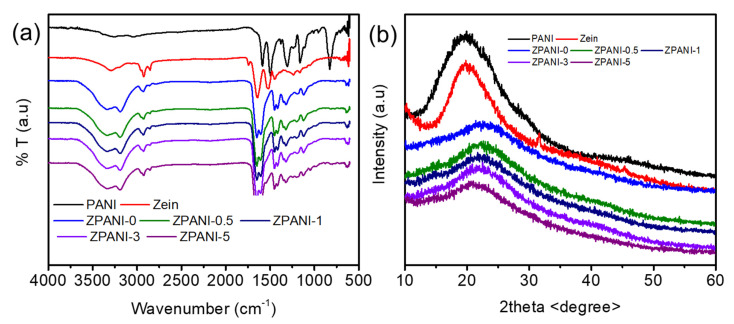
(**a**) FTIR spectra and (**b**) XRD patterns of ZPANI hydrogels.

The functional groups and crystallinity of ZPANI hydrogels were characterized based on FTIR and XRD patterns (Figure 5). The FTIR spectrum of polyaniline (PANI) reveals its characteristic structural features, providing insights into its chemical composition and bonding interactions (Figure 5a). The peaks at 1587 cm^−1^ and 1489 cm^−1^ correspond to the stretching vibrations of quinoid and benzenoid rings, respectively, which are integral to the conjugated structure of PANI, reflecting alternating single and double bonds between nitrogen and aromatic carbons. The band at 1309 cm^−1^ is attributed to C-N stretching in the aromatic amine, indicative of secondary amines that are crucial for PANI’s electrical conductivity. The peak at 1161 cm^−1^ corresponds to the in-plane bending vibrations of C-H bonds in the aromatic rings, characteristic of the protonated emeraldine salt form of PANI. Additionally, the absorption at 823 cm^−1^ represents out-of-plane C-H deformations, reflecting the substitution pattern in the aromatic rings. These FTIR features confirm the successful synthesis of PANI in its conductive emeraldine salt form, highlighting its conjugated structure, protonation, and stability. These properties, including conductivity and structural integrity, validate PANI’s potential for use in electronic devices, sensing applications, and energy-harvesting technologies, particularly when incorporated into hydrogels. The FTIR spectrum of the zein protein revealed two prominent bands in the amide I region at 1642 cm^−1^ and the amide II band at 1518 cm^−1^, which are characteristic of protein structures. A peak at 3289 cm^−1^ is attributed to –OH stretching. Additionally, a characteristic band at 1745 cm^−1^ corresponds to the C=O stretch associated with lipids. Upon crosslinking to form the hydrogel, the FTIR spectrum showed peaks at 1649 cm^−1^ and 1603 cm^−1^, which are assigned to the amide I and amide II bands of the crosslinked PAM network. The peaks corresponding to the amide groups in zein are now combined with the amide peaks of the PAM network, indicating the formation of interactions between zein and the crosslinked PAAm. The addition of PANI did not introduce new peaks or significantly alter the functional groups of the ZPANI hydrogel, suggesting that the incorporation of PANI did not disrupt the established interactions but may enhance the overall conductivity and mechanical properties of the hydrogel.

**Figure 6:**
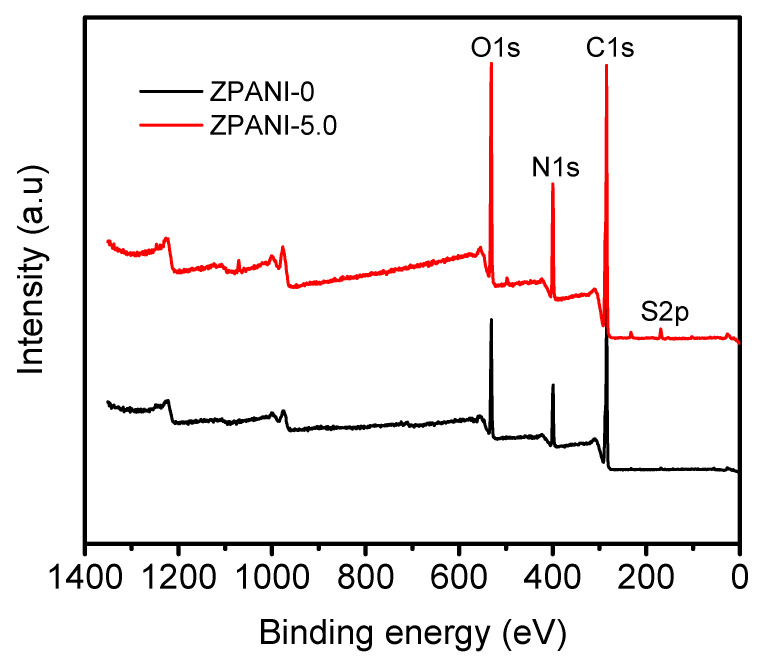
XPS spectra of the ZPANI-0 and ZPANI-5.0 hydrogels.

The XRD analysis revealed distinct structural changes in PANI, pure zein, and ZPANI hydrogels (Figure 5b). Pure PANI exhibited a semi-crystalline peak at 19.8°, reflecting its partial crystalline nature essential for electronic conductivity, while pure zein showed a sharp crystalline peak at 20°, attributed to the ordered packing of its molecules. Incorporating PANI into a zein hydrogel disrupted its crystalline structure, resulting in an amorphous peak at 22°, with an increasing PANI content further shifting this peak to lower angles, indicating enhanced molecular disorder. This structural transition improves the flexibility, conductivity, and hydrophilicity of the hydrogel, highlighting its potential for applications in sensors, bioelectronics, and tissue engineering. Further, the XPS survey scans of ZPANI-0 and ZPANI-5.0 hydrogels revealed significant differences in elemental composition, highlighting the incorporation of PANI into the zein-based hydrogel matrix (Figure 6). For ZPANI-0 (without PANI), the composition was 0.4% sulfur (S2p), 67.4% carbon (C1s), 14% nitrogen (N1s), and 18.2% oxygen (O1s), with the majority of carbon originating from the organic components of the zein matrix. In contrast, ZPANI-5.0, containing 5 wt% PANI, showed an increase in the sulfur (1.14%), nitrogen (15.37%), and oxygen (20.05%) content, while carbon decreased to 63.43%. The increase in the sulfur and nitrogen content is attributed to the presence of PANI, which contains nitrogen in its polymerized form. The oxygen content also increased due to the incorporation of oxygen-containing groups from both the crosslinking agent and PANI. These changes confirm the successful incorporation of PANI, which enhances the electrical conductivity and functional properties of the hydrogel, making it suitable for applications in sensors, energy harvesting, and bioelectronics. The XPS data support the intended chemical modification and demonstrates the effectiveness of PANI in improving the hydrogel’s properties.

### 2.5. Surface Morphology of ZPANI Hydrogels

**Figure 7:**
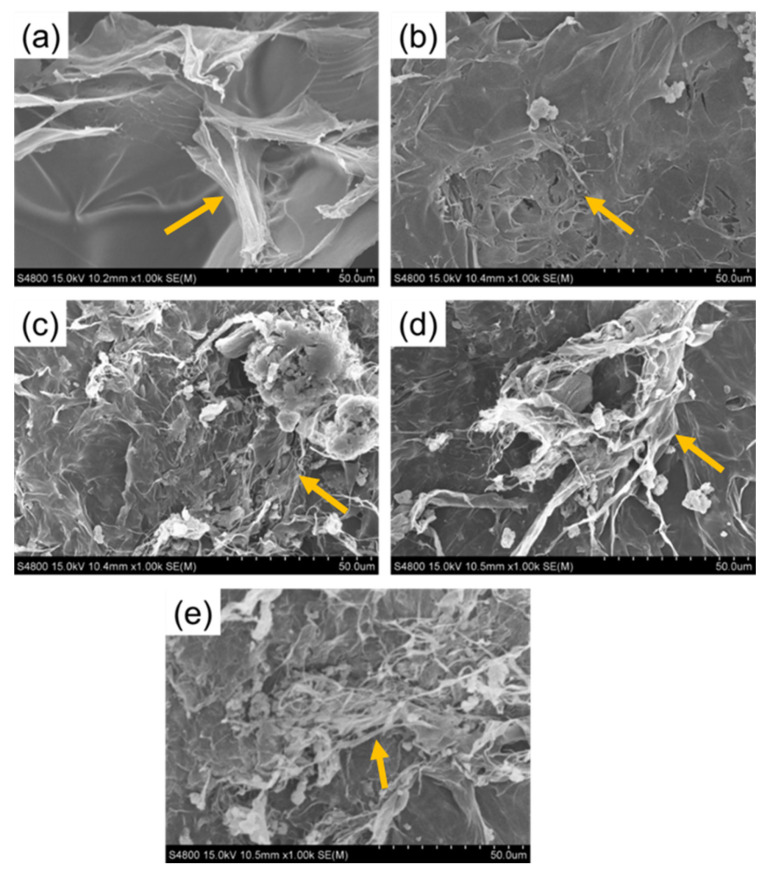
SEM images of (**a**) ZPANI-0, (**b**) ZPANI-0.5, (**c**) ZPANI-1.0, (**d**) ZPANI-3.0, and (**e**) ZPANI-5.0 hydrogels (arrow represent the fibrous nature of hydrogels).

The surface morphology of ZPANI hydrogels is showed in Figure 7. The ZPANI-0 hydrogel exhibits a sheet-like fibrous structure, which contributes to the adhesion properties of the hydrogel, making it suitable for attachment to various substrates. This fibrous morphology is advantageous for applications where the hydrogel needs to adhere to or interact with surfaces, such as in sensors or bioelectronics. Upon the incorporation of 0.5 wt% polyaniline (PANI), the hydrogel retains its fibrous structure but also begins to display aggregated nanostructures of PANI. This aggregation is likely due to the interaction of APS with PANI, which could lead to localized polymerization or crosslinking, promoting the formation of these nanostructured aggregates within the matrix. As the PANI content increases, the structure evolves further, with the fibrous PANI component becoming more prominent. At higher PANI concentrations (e.g., 3 wt% and 5 wt%), the hydrogel develops a more complex structure characterized by intertwined fibrous PANI networks alongside nanoaggregates. These nanostructures improve the mechanical properties of the hydrogel, increasing its surface area and potentially enhancing conductivity and strain sensing capabilities. The presence of both PANI fibers and nanoaggregates at higher concentrations of PANI (5 wt%) significantly enhances the mechanical integrity and structural functionality of the hydrogel. The aggregation of PANI leads to a denser, more interconnected network, which may offer better support for strain-sensing and energy-harvesting applications. This morphology could contribute to improved electrical properties, such as higher conductivity and an enhanced piezoelectric response, making the hydrogel more suitable for applications in flexible electronics, wearable sensors, and energy-harvesting devices. The unique combination of fibrous and nanoaggregate structures in the ZPANI hydrogels offers significant advantages in terms of the mechanical performance, surface interaction, and electrical characteristics, particularly as the PANI content increases.

### 2.6. Swelling Property of ZPANI Hydrogels

**Figure 8:**
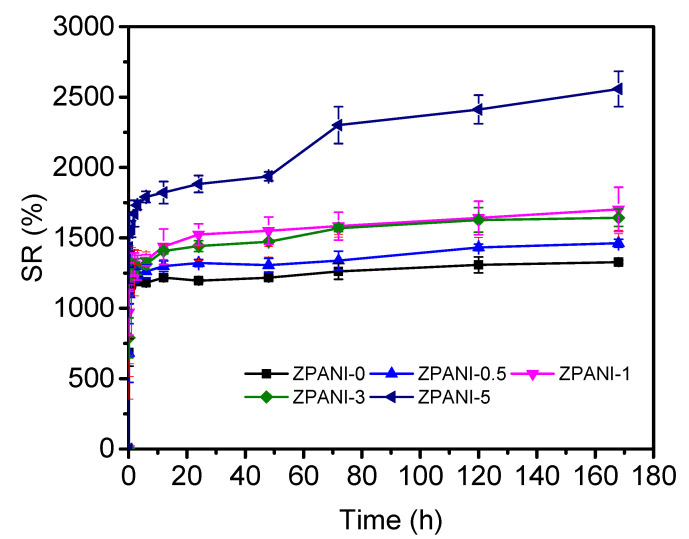
Swelling properties of ZPANI hydrogels.

The swelling behavior of zein-based hydrogels and their composites with varying amounts of PANI was evaluated to understand the impact of PANI incorporation on the water-retention capacity of the hydrogels (Figure 8). The zein hydrogel alone demonstrated a high swelling capacity, reaching an equilibrium swelling of 1200% within 12 h. This significant swelling is attributed to the hydrophilic nature of zein protein, which interacts with water molecules through hydrogen bonding, facilitating the rapid uptake of water and the expansion of the hydrogel network. Upon incorporating 0.5 wt% PANI into the zein hydrogel, the swelling capacity increased to 1300%, with equilibrium being reached within 24 h. The increase in swelling can be attributed to the conductive nature of PANI, which enhances the water-absorption ability of the hydrogel by altering the interaction between the hydrogel network and water molecules. The presence of PANI may introduce additional ionic groups and increase the hydrophilicity of the composite, allowing for the greater uptake of water. Further increases in the PANI content, from 1 to 3 wt%, did not result in significant changes in the swelling behavior, as the swelling reached similar values of around 1300%. This suggests that at low-to-moderate concentrations, the additional PANI did not substantially alter the swelling properties of the hydrogel. It is likely that the hydrogel network had already reached a point of optimal water-retention capacity, and additional PANI may not significantly contribute to further swelling. However, at a higher PANI concentration of 5 wt%, a substantial enhancement in swelling was observed, with the hydrogel reaching an equilibrium swelling of 2550% over a 14-day period. This drastic increase in swelling suggests that the higher PANI concentration significantly influenced the hydrogel’s ability to absorb and retain water. The addition of 5 wt% PANI may lead to a more open and flexible hydrogel network, which can accommodate more water molecules. The higher ionic content from PANI could also promote additional interactions with water, facilitating greater water retention. Overall, the swelling results highlight the importance of the PANI concentration in modulating the swelling behavior of zein-based hydrogels. At lower concentrations, PANI had a modest impact, but at higher concentrations, it dramatically enhanced the swelling capacity. This behavior suggests that the zein–PANI composite hydrogel could be tailored for applications requiring high swelling capacity and extended water retention, such as in drug delivery, wound healing, and tissue engineering.

### 2.7. Adhesion Properties of Hydrogels

The adhesion of a ZPANI hydrogel to skin or other substrates arises from multiple interactions, such as hydrophobic, electrostatic, hydrogen bonding, and van der Waals forces. The hydrogels (ZPANI-0 and ZPANI-5.0) showed good adhesiveness with various substrates (plastic, glass, wood, rubber, paper cup, glass, metal, spatuala, steel, PTFE, and pestle) (Figures S2 and S3). The ZPANI-0 hydrogel withstood 50 g, whereas, the ZPANI-5.0 hydrogel withstood a 100 g weight (Figure S4). Skin adhesion is crucial for wearable electronics, as the hydrogel must adhere to the skin, without the need for adhesive tapes, and maintain flexibility. To assess this, the hydrogel’s adhesion to porcine skin was tested. Hydrogels, such as ZPANI-0, ZPANI-0.5, ZPANI-1.0, ZPANI-3.0, and ZPANI-5.0, showed skin-adhesion strengths of 20.6 ± 1.3 kPa, 24.6 ± 1.6 kPa, 26.4 ± 1.1 kPa, 28.3 ± 1.1 kPa, and 30.1 ± 2.1 kPa, respectively. The skin-adhesion strength increased with a higher concentration of PANI. Incorporating PANI into a zein hydrogel enhances its adhesion to skin and other substrates through several mechanisms, including electrostatic, hydrogen bonding, hydrophobic, and π–π interactions. PANI’s conductive nature enables mechanoresponsive adhesion, adapting to substrate contours and maintaining contact under pressure, which is ideal for wearable applications. Its structural rigidity reinforces the hydrogel matrix while preserving flexibility, promoting a tight surface seal and resistance to mechanical stresses. Additionally, PANI’s molecular structure facilitates van der Waals interactions and improves interfacial compatibility, while displacing water at moist surfaces, like the skin, allowing for stronger hydrogen bonding and cohesive adhesion. This combination results in a robust and durable adhesive matrix suitable for flexible, high-performance applications.

### 2.8. In Vitro Biocompatibility Analysis

**Figure 9:**
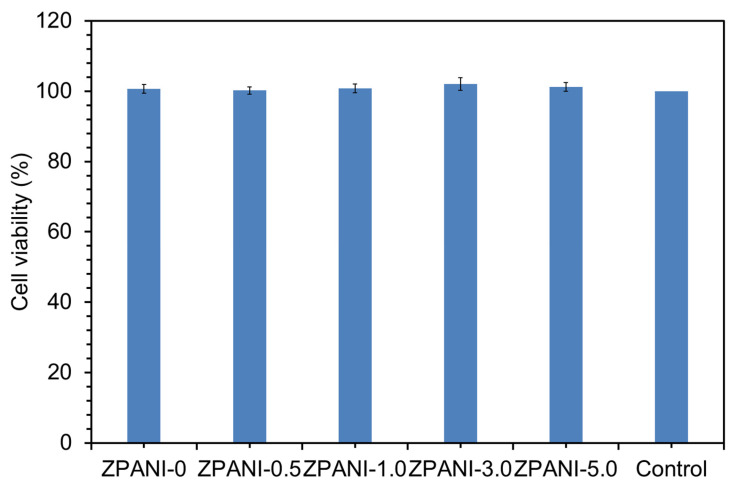
In vitro biocompatibility was studied in terms of cell viability calculated using Prestoblue cell viability assays for ZPANI hydrogels treated with NIH 3T3 cells for a 72 h incubation.

In addition to the skin-adhesion properties of the hydrogels, an analysis of their biocompatibility is crucial for wearable strain sensor applications, as it ensures both safe and effective integration with the human body for prolonged use without causing adverse reactions [19]. The biocompatibility of ZPANI composite hydrogels was assessed to determine their potential for biomedical applications. Skin fibroblast cells (NIH3T3) were cultured on the hydrogels to evaluate their ability to support cell growth and proliferation (Figure 9). The hydrogels were sterilized and incubated with fibroblast cells for 72 h, and the Prestoblue assay was conducted to measure the metabolic activity and viability of the cells. The results indicated that all ZPANI hydrogel formulations exhibited excellent cytocompatibility, with 100% cell viability, suggesting that the hydrogels did not exert any significant cytotoxic effects even with varying concentrations of PANI. Based on these results, the ZPANI composite hydrogels demonstrate strong potential for use in tissue engineering, wound healing, and bioelectronics, thanks to their excellent cell viability, adhesion properties, and favorable cytocompatibility. These hydrogels can serve as bioactive scaffolds for tissue regeneration and as skin wound healing materials, where effective tissue adhesion and minimal irritation are critical. The strong performance of the ZPANI hydrogels in terms of both biocompatibility and mechanical properties also makes them suitable candidates for applications in wearable strain sensors. The hydrogels’ combination of mechanical integrity, conductivity, and biocompatibility offers a promising avenue for advanced healthcare and electronic applications.

### 2.9. Mechanoresponsive Energy Harvesting

The conductivity of the zein–polyacrylamide hydrogels reinforced with varying amounts of PANI was assessed using Electrochemical Impedance Spectroscopy (EIS) (Figure S5). The addition of PANI significantly enhanced the electrical conductivity of the hydrogels [20,21]. The trend indicates that PANI incorporation leads to a significant improvement in the conductivity of the hydrogels, with the highest conductivity observed for the ZPANI-5.0 sample. The increase in conductivity with the addition of PANI to the zein–polyacrylamide hydrogel matrix is attributed to the conductive nature of PANI. As a conductive polymer, PANI introduces additional pathways for electron transfer within the hydrogel network. At low concentrations (0.5% and 1.0%), PANI effectively enhances the hydrogel’s electrical conductivity by creating conductive networks without significantly altering the hydrogel’s overall structure. As the PANI content increases, the hydrogel’s conductivity continues to rise, with the highest conductivity observed in the ZPANI-5.0 sample, which suggests a more extensive network of conductive PANI chains within the matrix.

**Figure 10:**
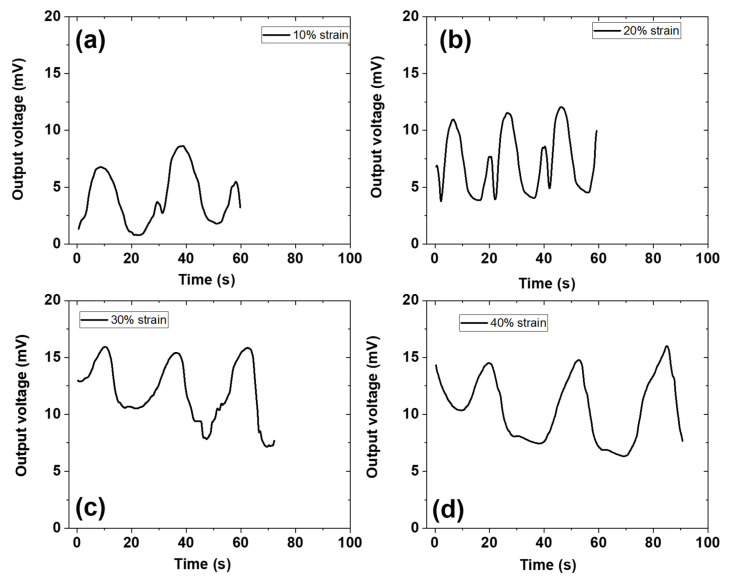
Mechanoresponsive output energy signal generation of ZPANI-5.0 under (**a**) 10% strain, (**b**) 20% strain, (**c**) 30% strain, and (**d**) 40% strain.

The electromechanical behavior of the ZPANI-5.0 hydrogel, with 5 wt% PANI, was tested under increasing compressive strain, ranging from 10% to 40% (Figure 10). The hydrogel showed an increase in the output voltage as the compressive strain increased, with a steady rise in electrical conductivity. The voltage output continued to increase up to a compressive strain of 30%, after which it remained constant. This behavior is indicative of the hydrogels’ piezoelectric response, where the increased compressive strain leads to a higher output voltage within 20 s of response time, thereby enhancing the hydrogel’s ability to function as a strain sensor. At a higher compressive strain, the hydrogel’s ability to store and release energy also becomes evident, contributing to its energy-harvesting potential. The increase in voltage with strain is attributed to the enhanced conductivity of the polyaniline component, which facilitates the piezoelectric response and energy conversion. The constant output voltage at higher strains further suggests that the hydrogel maintains stability and durability under continuous deformation, a critical requirement for wearable sensors that must endure repetitive mechanical stress. The combination of PANI’s excellent conductivity, the flexible nature of zein, and the crosslinking of the PAM network creates a robust material with versatile applications in wearable electronics. The ZPANI hydrogels exhibit both mechanical strength and viscoelasticity, which are necessary for maintaining stability under various strain conditions. The enhanced strain-sensing capabilities, coupled with the ability to harvest and store energy, make these hydrogels suitable for next-generation healthcare applications. The biocompatibility of the hydrogels, confirmed through cell culture assays, further supports their potential for biomedical applications, such as continuous health monitoring and electronic skin devices.

**Figure 11:**
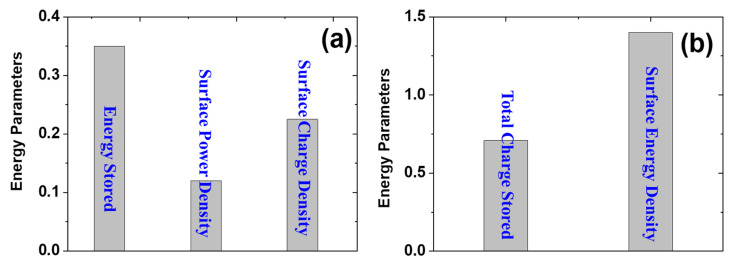
Energy related parameters of ZPANI-5.0 hydrogel (**a**) Energy stored, surface power density, and surface charge density and (**b**) total charge stored and surface energy density.

One of the key applications for the synthesized PANI is in energy harvesting. The incorporation of PANI into the zein composite hydrogel has shown promising results in energy harvesting and wearable sensor applications. Energy harvesting assessments of the ZPANI-5.0 hydrogels revealed a surface power density of 0.12 nW cm^−2^, energy storage of 0.35 nJ, total charge storage of 0.71 nJ, surface charge density of 0.225 nJ cm^−2^, and surface energy density of 1.4 pWh cm^−2^ (Figure 11). These values highlight the efficient energy-conversion capabilities of the ZPANI hydrogels, making them ideal candidates for wearable devices that require low power generation and energy storage, such as health monitoring systems and bioelectronics.

## 3. Conclusions

Tissue-adhesive ZPANI hydrogels exhibit outstanding properties, positioning them as promising candidates for next-generation wearable strain sensors and mechanoresponsive energy-harvesting applications. The incorporation of polyaniline (PANI) significantly enhances electrical conductivity, while the hydrogels’ swelling behavior ensures flexibility and strong adhesion to various substrates, including biological tissues. Comprehensive structural and chemical analyses confirm their composition and surface characteristics, while biocompatibility studies validate their safety for biomedical applications. Mechanical and rheological assessments reveal a trade-off between mechanical strength and electrical conductivity, highlighting the need for an optimized PANI content to balance the strain-sensing performance, viscoelasticity, and durability. Despite reduced mechanical strength at higher PANI concentrations, the hydrogels exhibit robust voltage output and energy-harvesting capabilities, demonstrating their feasibility for self-powered wearable electronics. These findings underscore the potential of ZPANI hydrogels in bioelectronic applications, including real-time health monitoring and flexible energy-harvesting devices. Future work should focus on further optimizing their composition, enhancing long-term stability, and exploring integration with advanced bioelectronic platforms to maximize their practical utility.

## 4. Materials and Methods

### 4.1. Materials

Zein, aniline (ANI), sodium dodecyl sulphate (SDS) ammonium persulfate (APS), N,N-methylene-bis (acrylamide) (BIS), and N,N,N,N-Tetramethylethylenediamine (TEMED) were purchased from Sigma-Aldrich, Seoul, Republic of Korea. Acrylamide (AM) was purchased from Dae-Jung chemical metal Co., Ltd., Siheung-si, Gyeonggi-Do, Republic of Korea. Polyaniline (PANI) was synthesized using ammonium persulfate (APS) as an initiator in an acidic medium [20]. The ANI monomer (1.0 mL) was dissolved in 50 mL of 1 M HCl, and APS (2.28 g) was dissolved separately in 50 mL of 1 M HCl. The APS solution was added dropwise to the aniline solution under constant stirring at 0–5 °C, maintaining controlled conditions for polymerization. The reaction mixture was stirred for 6 h, turning dark green, indicating the formation of PANI in its emeraldine salt form. The precipitate was filtered, washed with deionized water and ethanol, and dried at 50 °C for 12 h.

### 4.2. Preparation of ZPANI Hydrogels

To prepare the ZPANI composite hydrogels, a 12 mL solution of zein (5 wt% in SDS) was prepared and mixed with varying amounts of PANI (0, 0.5, 1, 3, and 5 wt%, based on the weight of the acrylamide monomer). Subsequently, 2.0 g of acrylamide, 5 mg of MBA, and 200 mg of APS were added sequentially with thorough mixing after each addition. Finally, 10 µL of TEMED was added to initiate gelation. The reaction mixtures were poured into molds and allowed to form hydrogels within 10 min at room temperature. The hydrogels were then left undisturbed for an additional 6 h to ensure complete crosslinking and stabilization. The hydrogels without PANI were also prepared using the same procedure. The hydrogels were designated, based on the PANI content, as ZPANI-0, ZPANI-0.5, ZPANI-1.0, ZPANI-3.0, ZPANI-5.0, and ZPANI-7.0 corresponding to 0, 0.5, 1, 3, 5, and 7 wt% of PANI, respectively.

### 4.3. Characterization

The chemical structure and functional groups of the ZPANI hydrogels were analyzed using a Perkin Elmer FTIR spectrometer, with measurements conducted across the wavenumber range of 4000–500 cm^−1^. This analysis provided insights into the interactions among zein, polyaniline (PANI), and the hydrogel network components. The crystallinity and structural organization of the ZPANI hydrogels were studied using a Bruker AXS D8 Advance X-ray diffractometer in Bragg–Brentano geometry, utilizing Cu Kα radiation (wavelength ~1.54 Å). The samples were mounted on a rotating stage for precise alignment, and diffraction patterns were recorded to identify crystalline and amorphous regions. The surface chemical composition and elemental states were analyzed using X-ray photoelectron spectroscopy (XPS, Thermo Scientific K-Alpha+, Waltham, MA, USA), with high-resolution spectra recorded to identify the chemical states of elements present in the hydrogel matrix and to confirm the incorporation of polyaniline. The morphology of the freeze-dried ZPANI hydrogels was analyzed using field emission scanning electron microscopy (FE-SEM), operated at an acceleration voltage of 5.0 kV. Prior to imaging, the hydrogel surfaces were sputter-coated with platinum at a low deposition rate to enhance conductivity and prevent charging effects. Tensile properties were measured using a universal testing machine (MCT 2150, A&D Co., Ltd., Tokyo, Japan). Cylindrical hydrogel samples (10 mm diameter × 10 mm length) were subjected to a 5 kN load at a constant speed of 50 mm/min to determine the tensile strength, strain, and Young’s modulus. Compression testing was also conducted using the same machine under identical conditions to evaluate the hydrogel’s capacity to withstand compressive forces.

### 4.4. Swelling Properties

To assess the swelling behavior of the ZPANI composite hydrogels, the dry weight (W_d_) of each hydrogel sample was determined prior to immersion. The hydrogels were immersed in 10 mL of DDW solution and incubated at room temperature. The swelling was monitored at predetermined time intervals. At each time point, the swollen hydrogel samples were removed, gently blotted to remove excess DDW, and weighed to determine the wet weight (W_s_). The percent swelling ratio (% SR) was calculated using the following formula:

$$\%SR=\frac{W_{s}-W_{d}}{W_{d}}\times 100$$

where W_s_ is the weight of the swollen hydrogel and W_d_ is the dry weight of the hydrogel. Three samples (N = 3) were tested for each time point to ensure reproducibility and accuracy. This procedure provides insights into the water-absorption capacity and the swelling kinetics of the hydrogels over time.

### 4.5. Adhesive Properties

To evaluate the adhesive properties of the ZPANI composite hydrogels, a tensile test was performed using a universal testing machine (MCT 2150 tensile tester (A&D Co., Ltd., Tokyo, Japan)), as described in previous studies. The hydrogels were applied to the surface of porcine skin with a bonding area of 20 × 20 mm^2^. The hydrogels were firmly pressed onto the skin to ensure proper adhesion. The maximum adhesion strength was determined by applying a tensile force until detachment occurred. The adhesive strength was calculated as the maximum load (N) at failure divided by the bonded area (in mm^2^). This method provides an understanding of the hydrogels’ ability to adhere to biological tissues, which is critical for their potential use in wound healing or biomedical applications.

### 4.6. Biocompatibility Analysis

To evaluate the biocompatibility of the ZPANI composite hydrogels, the Prestoblue assay was performed using skin fibroblast cells (NIH_3_T_3_) American Type Culture Collection (ATCC) (Manassas, VA, USA). The hydrogels were first sterilized in ethanol, washed with phosphate-buffered saline (PBS), and fixed in 24-well plates with Dulbecco’s Modified Eagle Medium (DMEM). Skin fibroblast cells (5 × 10^4^ cells) were seeded in each well and incubated for 72 h. After the incubation period, the media were removed, and 100 µL of Prestoblue solution (1:10 dilution) was added to each well and incubated for an additional 2 h. The optical density (OD) of the samples was measured at 570 and 600 nm using a microplate reader to assess cell viability. The viability was calculated by normalizing the average OD of the hydrogel-treated cells to that of the control group.

### 4.7. Electrochemical Impedance Spectroscopy (EIS)

EIS was conducted using an electrochemical workstation (Biologica) across a frequency range of 0.1–105 Hz. The cylindrical hydrogels were placed between metal plates and linked to an electrochemical workstation (Corrtest-CS250) for the EIS measurements.

### 4.8. Mechanoresponsive Output Voltage Measurements

The output voltage of the ZPANI-5.0 hydrogel was assessed under compressive mechanical strain using a universal testing machine (UTM) (MCT 2150 tensile tester (A&D Co., Ltd., Tokyo, Japan)) and a multimeter (Model 34401A, Agilent Technologies Inc.; Santa Clara, CA, USA). The voltage response was recorded for about 1 min while applying compressive strains ranging from 10 to 40% at a strain rate of 4 mm/min.

## Data Availability

The original contributions presented in this study are included in the article/Supplementary Materials. Further inquiries can be directed to the corresponding authors.

## Supplementary Materials

The following supporting information can be downloaded at: https://www.mdpi.com/article/10.3390/gels11050307/s1, Figure S1: SEM image of PANI (a) low resolution and (b) high resolution; Figure S2: Adhesion of ZPANI-0 hydrogel on various substrates; Figure S3: Adhesion of ZPANI-5.0 hydrogel on various substrates; Figure S4: Adhesion of ZPANI-0 hydrogel on plastic tube filled with 50 g water, (b) ZPANI-5.0 on plastic tube filled with 50 g, and (c) ZPANI-5.0 hydrogel adhesion with 100 g steel; Figure S5: EIS of hydrogels.

## References

[1] Wang X., Liu Z., Zhang T. (2017). Flexible sensing electronics for wearable/attachable health monitoring. Small.

[2] Khan S., Ali S., Bermak A. (2019). Recent developments in printing flexible and wearable sensing electronics for healthcare applications. Sensors.

[3] Niu Y., Liu H., He R., Li Z., Ren H., Gao B., Guo H., Genin G.M., Xu F. (2020). The new generation of soft and wearable electronics for health monitoring in varying environments: From normal to extreme conditions. Mater. Today.

[4] Zhao J., Feng S., Cao X., Zheng H. (2024). Wearable sensors for monitoring vital signals in sports and health: Progress and perspective. Sens. Rev..

[5] Li Y., Gu Y., Teng J., Zheng S., Pang Y., Lu X., Liu B., Liu S., Zhao Q. (2024). Advancing EEG-based brain-computer interface technology via PEDOT: PSS electrodes. Matter.

[6] Liang X., Zhong H.J., Ding H., Yu B., Ma X., Liu X., Chong C.M., He J. (2024). Polyvinyl alcohol (PVA)-based hydrogels: Recent progress in fabrication, properties, and multifunctional applications. Polymers.

[7] Wu Y., Luo Y., Cuthbert T.J., Shokurov A.V., Chu P.K., Feng S.P., Menon C. (2022). Hydrogels as soft ionic conductors in flexible and wearable triboelectric nanogenerators. Adv. Sci..

[8] Zheng S., Chen X., Shen K., Cheng Y., Ma L., Ming X. (2024). Hydrogen bonds reinforced ionogels with high sensitivity and stable autonomous adhesion as versatile ionic skins. ACS Appl. Mater. Interfaces.

[9] Zhang Y. (2020). Stimuli-Responsive Microgel-Based Materials and Their Assembly for Controlled Drug Delivery and Sensing Systems. Ph.D. Thesis.

[10] Cui C., Fu Q., Meng L., Hao S., Dai R., Yang J. (2020). Recent progress in natural biopolymers conductive hydrogels for flexible wearable sensors and energy devices: Materials, structures, and performance. ACS Appl. Bio Mater..

[11] Han F., Chen S., Wang F., Liu M., Li J., Liu H., Yang Y., Zhang H., Liu D., He R. (2025). High-Conductivity, Self-Healing, and Adhesive Ionic Hydrogels for Health Monitoring and Human-Machine Interactions under Extreme Cold Conditions. Adv. Sci..

[12] Jaski A.C., Schmitz F., Horta R.P., Cadorin L., da Silva B.J.G., Andreaus J., Paes M.C.D., Riegel-Vidotti I.C., Zimmermann L.M. (2022). Zein—A plant-based material of growing importance: New perspectives for innovative uses. Ind. Crops Prod..

[13] Yan X., Li M., Xu X., Liu X., Liu F. (2022). Zein-based nano-delivery systems for encapsulation and protection of hydrophobic bioactives: A review. Front. Nutr..

[14] Berradi A., Aziz F., Achaby M.E., Ouazzani N., Mandi L. (2023). A comprehensive review of polysaccharide-based hydrogels as promising biomaterials. Polymers.

[15] Pérez-Guzmán C.J., Castro-Muñoz R. (2020). A review of zein as a potential biopolymer for tissue engineering and nanotechnological applications. Processes.

[16] Zubair M., Hussain S., Hussain A., Akram M.E., Shahzad S., Rauf Z., Mujahid M., Ullah A. (2024). Trends in protein-derived materials for wound care applications. Biomater. Sci..

[17] Pyarasani R.D., Jayaramudu T., John A. (2019). Polyaniline-based conducting hydrogels. J. Mater. Sci..

[18] Han X., Xiao G., Wang Y., Chen X., Duan G., Wu Y., Gong X., Wang H. (2020). Design and fabrication of conductive polymer hydrogels and their applications in flexible supercapacitors. J. Mater. Chem. A.

[19] Zarei M., Lee G., Lee S.G., Cho K. (2023). Advances in biodegradable electronic skin: Material progress and recent applications in sensing, robotics, and human–machine interfaces. Adv. Mater..

[20] Wu Y., Chen Y.X., Yan J., Quinn D., Dong P., Sawyer S.W., Soman P. (2016). Fabrication of conductive gelatin methacrylate–polyaniline hydrogels. Acta Biomater..

[21] Reza M., Srikandi N., Amalina A.N., Benu D.P., Steky F.V., Rochliadi A., Suendo V. (2019). Variation of ammonium persulfate concentration determines particle morphology and electrical conductivity in HCl-doped polyaniline. IOP Conf. Ser. Mater. Sci. Eng..

