# Supplementary material for: Tissue-Adhesive and Biocompatible Zein-Polyaniline-Based Hydrogels for Mechanoresponsive Energy-Harvesting Applications
Source: Gels. 2025 Apr 22;11(5):307. doi: 10.3390/gels11050307 (PMC12111734; doi:10.3390/gels11050307)
Supplement: Supplementary file 1 [file gels-11-00307-s001.zip › gels-3511871-supplementary.pdf]

Supporting Information

Article

Tissue-Adhesive and Biocompatible Zein-Polyaniline-Based Hydrogels for Mechanoresponsive Energy Harvesting Applications

Maduru Suneetha [1*], Seainn Bang,[1] Sarah A. Alshehri[2] and Sung Soo Han[1 & 3,*]

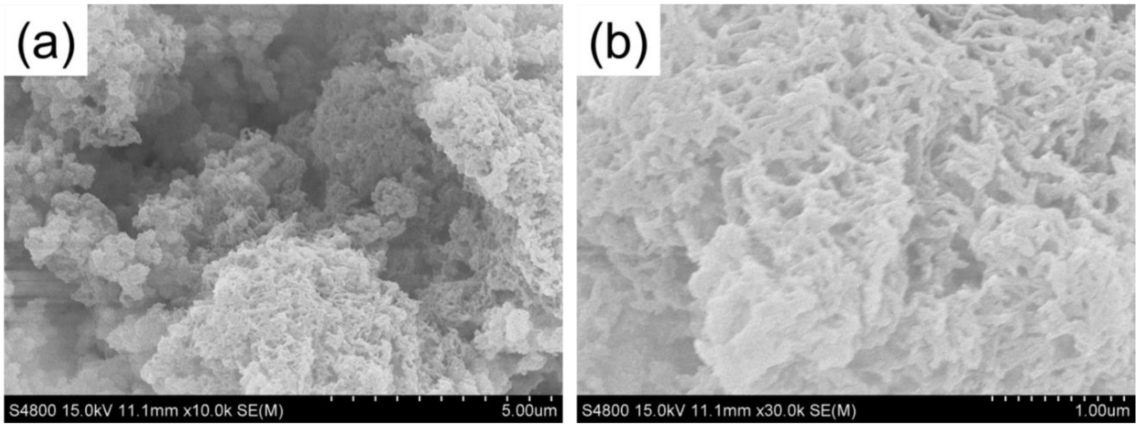

Figure S1. SEM image of PANI (a) low resolution and (b) high resolution.

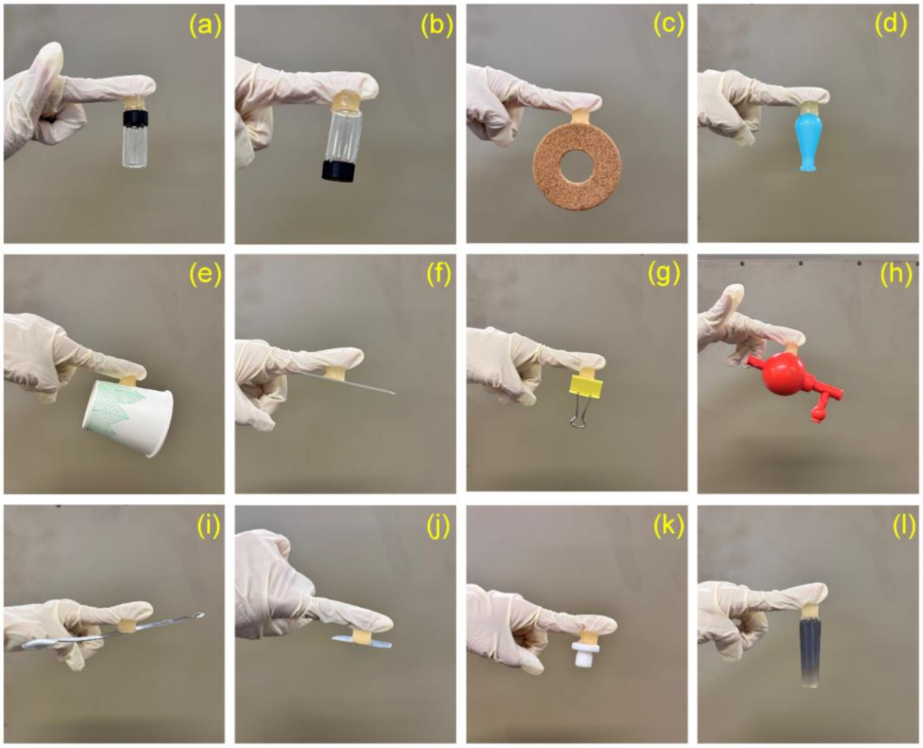

Figure S2. Adhesion of ZPANI-0 hydrogel on various substrates.

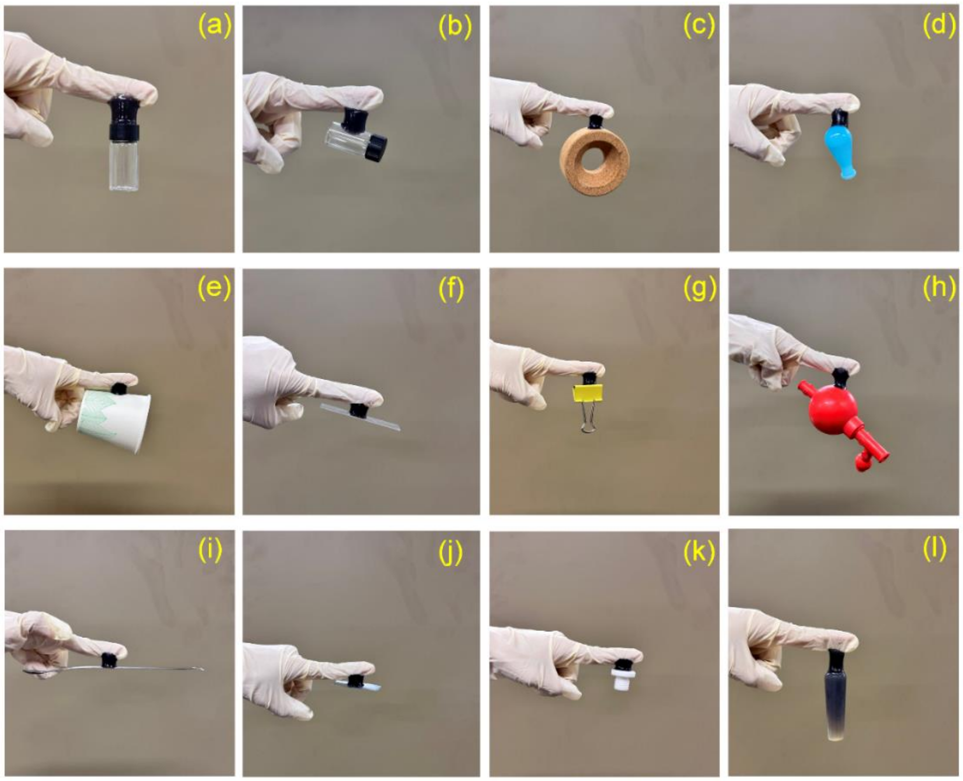

Figure S3. Adhesion of ZPANI-5.0 hydrogel on various substrates.

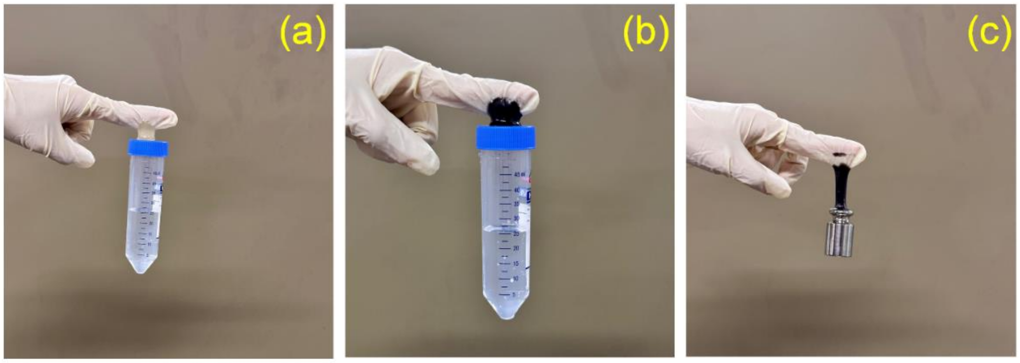

Figure S4. Adhesion of ZPANI-0 hydrogel on plastic tube filled with 50 g water, (b) ZPANI-5.0 on plastic tube filled with 50 g, and (c) ZPANI-5.0 hydrogel adhesion with 100 g steel.

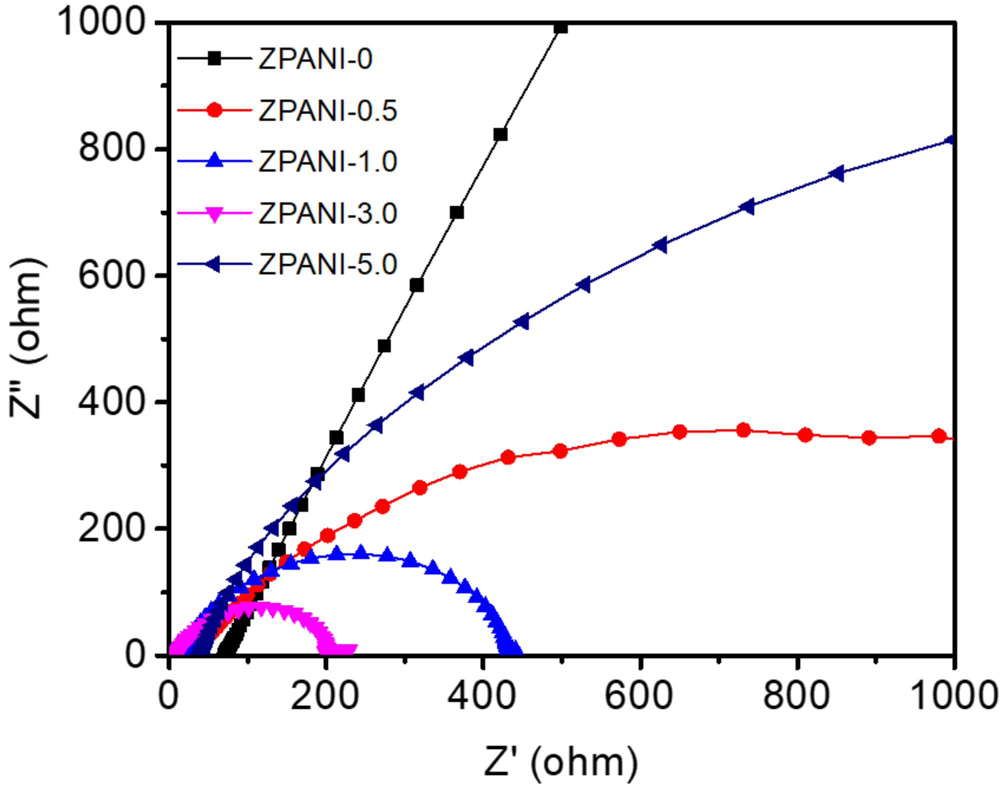

Figure S5. EIS of hydrogels
